# Using discrete event simulation to compare the performance of family health unit and primary health care centre organizational models in Portugal

**DOI:** 10.1186/1472-6963-11-274

**Published:** 2011-10-15

**Authors:** André S Fialho, Mónica D Oliveira, Armando B Sá

**Affiliations:** 1 Engineering Systems Division, Massachusetts Institute of Technology, 77 Massachusetts Avenue, 02139 Cambridge, MA, USA; 2 CEG-IST, Centre for Management Studies of Instituto Superior Técnico, Technical University of Lisbon, Av. Rovisco Pais, Lisbon 1049-001, Portugal; 3 Instituto de Medicina Preventiva, Faculdade de Medicina da Universidade de Lisboa, Av. Prof. Egas Moniz, Lisbon 1649-028, Portugal

**Keywords:** Family health units, primary care reforms, discrete event simulation, performance indicators, Portugal

## Abstract

**Background:**

Recent reforms in Portugal aimed at strengthening the role of the primary care system, in order to improve the quality of the health care system. Since 2006 new policies aiming to change the organization, incentive structures and funding of the primary health care sector were designed, promoting the evolution of traditional primary health care centres (PHCCs) into a new type of organizational unit - family health units (FHUs). This study aimed to compare performances of PHCC and FHU organizational models and to assess the potential gains from converting PHCCs into FHUs.

**Methods:**

Stochastic discrete event simulation models for the two types of organizational models were designed and implemented using Simul8 software. These models were applied to data from nineteen primary care units in three municipalities of the Greater Lisbon area.

**Results:**

The conversion of PHCCs into FHUs seems to have the potential to generate substantial improvements in productivity and accessibility, while not having a significant impact on costs. This conversion might entail a 45% reduction in the average number of days required to obtain a medical appointment and a 7% and 9% increase in the average number of medical and nursing consultations, respectively.

**Conclusions:**

Reorganization of PHCC into FHUs might increase accessibility of patients to services and efficiency in the provision of primary care services.

## Background

### General information

Primary health care services of good quality are widely recognized as critical for the improvement of health care systems [1-3], in particular when health care systems are being challenged by aging populations, an increased prevalence of chronic diseases, complexities of team-based contemporary practice and limited funding. Policy makers have been experimenting with different models of primary care delivery in order to enhance comprehensiveness, integration and accessibility [4]. In Portugal a major reform of primary health care was started in 2006 in order to address those challenges.

The Portuguese National Health Service (NHS) is defined as universal, nearly free at the point of use and funded by general taxation [5]. Although there are overlapping health systems (health insurance schemes from employer organizations and private health insurance plans) and a high use of private services in some specialties, most primary care services are provided by the NHS [6]. Primary care represents the first level of contact with the health system: general practitioners/family doctors (GPs) act both as care providers and gatekeepers to secondary care. Patients must register with a GP from a health centre, preferably in their area of residence, so they can be entitled to home visits if the need arises.

In 2005, primary care services were provided in 351 Primary Health Care Centers (PHCCs) and most of these (254) provided 12 or 24-hour health services for acute illness and some emergency care [7]. In the same year, on average, each PHCC had 30388 registered patients, employed 20 GPs and 20 nurses, and provided 75000 consultations, with an average of 1497 registered patients per GP. An additional amount of non-registered patients (on average 3376, approximately 10% of the total number of patients) sought care in PHCCs. An average of 15000 urgent or emergency consultations per PHCC was performed in that same year [7].

Until 2005, PHCCs had little autonomy and were depending on Regional Health Administrations for relevant management decisions [8]. At the time several problems in the primary care system were acknowledged. These problems included (i) small and inadequate numbers of GPs in some regions, together with the retirement of a growing number of GPs; (ii) high number of people not registered with a GP (10.6% according to [7]); (iii) high dissatisfaction of patients and doctors with primary care provision; and (iv) unnecessary overuse of hospital emergency services related to difficult access to primary care services [9]. In 1998 an experimental organizational model was launched by the Health Ministry, and 20 groups of GPs organized themselves in small autonomous functional units inside the existing health centres. The payment system included a capitation fraction. The evolution of these groups was closely monitored by the Ministry of Health, and relevant efficiency gains were identified [9,10]. This observation led to the development, in 2006, of a new organizational model for primary health care units, which were designated *family health units *(FHUs) [11]. These new units loosely resemble family health networks in Canada [12] and British practices. In PHCCs, health care centres operate under a rigid chain of command and control, and professionals are employed as civil servants through a complicated bureaucratic process. FHUs, on the other hand, are self-organized multi-professional teams formed by GPs, nurses, managers and other professionals. These teams have the autonomy to define their own working processes and to negotiate goals to be met with local health authorities [13]. A new payment system was set for these units.

Some differences between the PHCC and FHU organizational models should be highlighted for a better understanding of the changes involved. In some PHCCs acute cases are treated in separate facilities, staffed by the GPs of the PHCC, with opening hours varying between 3 and 24 hours, depending on the location of the PHCC; in FHUs acute cases are treated by GPs during their normal working hours. In most PHCCs only medical consultations are scheduled; in FHUs regular nursing appointments must also be scheduled. In PHCCs a fixed salary is the norm; in some FHUs (known as *model B FHUs*) remuneration is compounded by a smaller fixed salary fraction plus a series of supplements: (i) capitation (up to a defined ceiling); (ii) a complement for the provision of specific services under contract beyond the basic job description; (iii) a premium for achieving negotiated goals; and (iv) fee-for-service payments for house calls [14]. Many FHUs adopted model B as their remuneration system. In this paper, we focus on the FHU with a model B remuneration system.

By the end of 2010 299 FHUs were in place and in 2011 78 new ones are opening [15]. FHUs are expected to (i) increase motivation and satisfaction of both patients and health care professionals, (ii) outperform PHCCs in terms of quality, access and efficiency [8,11], and (iii) implicitly confirm the importance of team building and collaboration in the delivery of primary care services [16]. The FHU organizational model brought major changes in appointment scheduling, acute care delivery and staff payment system [17,18].

FHUs show a positive impact in preliminary evaluations of the reform process. According to Campos [9], these positive signs started to become visible at the end of 2007, with decreased demand of out-of-hours appointments, better doctor-patient relationship and higher degrees of satisfaction and motivation both from patients and professionals. In two complementary studies, Gouveia et al. [19,10] analysed the cost differences between PHCCs and FHUs, using econometric analysis. Results from those two studies show that, in spite of a higher level of GPs' remuneration in FHUs, global costs were lower in FHUs due to comparatively lower costs in key components of health care, such as on the spending of diagnostic tests, drugs and other procedures. However, and as far as we know, no studies compared the impact of FHUs on accessibility and efficiency. Also, the effect of converting PHCCs into FHUs has not been quantified. Most studies evaluating health care reforms in other countries did not quantify the impact of new organizational models and, when they did, they mostly analysed the impact on a narrow range of indicators.

### Simulation modelling in health care

Health care simulation models typically attempt to provide support for better operational decision making and planning [20]. Several health care administrators used discrete event simulation (DES) models as effective tools for allocating resources in the improvement of patient flow, while reducing health care delivery costs and increasing patient satisfaction [21]. The choice of a DES model to model PHCCs and FHUs is justified by its capacity for reproducing a systems' behaviour through the modelling of the relation between the inputs of a primary care delivery system (including patients, doctors, nurses, patient scheduling and patient routing) and various outputs measures (e.g. waiting times, patient throughput, staff utilization) [22]. Systems simulated with DES models consist of discrete entities which occupy discrete states that will change over time. Adapting examples from Pidd [23], a primary care centre will include individual patients who are entities and whose states may include 'being admitted', 'waiting for a consultation', 'being in a consultation with doctor', and so on. An entity is thus an object whose behaviour within the model will be explicitly tracked as the simulation proceeds. Similarly, doctors and nurses who treat the patients may themselves be regarded as entities that change state. Hence, a DES model aims to capture the important features of the system in terms of entities and states. DES models have a time dimension as entities change state through time; have activities (e.g. consultations) that may require the co-operation of more than a single class of entity (e.g. between doctors and patients); and contain processes in the form of a chronological sequence of activities through which an entity must or may pass (e.g. patients pathways). Each class of entity (e.g. patients of types A and B) will have one or more processes associated with it (e.g. will have associated different pathway(s)), and when an entity that is a member of a specific class (e.g. a single patient A) appears in the simulation, each process becomes a route through which the entity will pass (e.g. single patient A will pass through its associated pathway(s)). Thus, building a DES model needs a set of logical statements, expressed in a computable form, describing how the entities change state. Another feature of DES models, being stochastic simulation models, is that they allow the accounting for uncertainty in the demand and delivery of health care (e.g. in the time a patient spends in a consultation). A detailed explanation of DES models can be found elsewhere [23,24].

Despite the potential of DES models to analyse quality and efficiency improvements in health care systems [25], we did not find any studies using DES to evaluate organizational reforms in the primary care sector. Most simulation and DES studies were developed at the micro level, mainly focusing on the problems of scheduling and capacity planning (available reviews of DES models can be consulted in [21,20,25]). These studies seldom modelled whole health care units [25].

Few studies compared the performance of PHCCs and FHUs and, to the best of our knowledge, no study assessed the impact of converting PHCCs into FHUs. In this work, we used DES to model PHCC and FHU organizational models and to analyse the impact of expanding current primary care reforms in Portugal. We developed DES models to compare the impacts of adopting the PHCC or FHU organizational models on accessibility, productivity and costs and assessed the gains that could potentially be achieved with FHU adoption. This paper brings attention to the usefulness of DES models in the evaluation of organizational models and to the potential impacts of expanding the present Portuguese primary care reform.

## Methods

Two templates of DES models, for PHCCs and FHUs, were designed and implemented in the Simul8 computer software package (version 13.0) [26]. These two templates were applied and validated to a sample of PHCCs and FHUs from the Greater Lisbon area. Finally, the conversion of PHCCs into FHUs was modelled.

### Conceptual models

**Figure 1:**
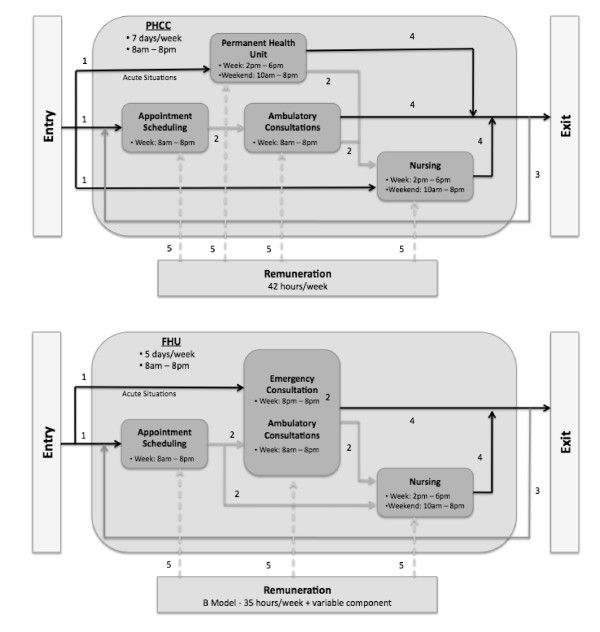
**Top: Conceptual model of a PHCC; Bottom: Conceptual model of an FHU**. (1) Patients entering the health care unit, (2) internal flows of patients inside the unit, (3) patients re-entering the system for follow-up consultations, (4) exit of patients from the unit and (5) the model's specific remuneration type.

We started by representing two conceptual models that included the processes and flows displayed in Figure 1. Flows are shown by five different sets of arrows that represent: (i) patients entering the health care unit, (ii) internal flows of patients inside the unit, (iii) patients re-entering the system for follow-up consultations, (iv) exit of patients from the unit and (v) the model's specific remuneration type.

Four types of consultations were modelled in both types of organizational units - medical, emergency/acute and nursing type 1 and type 2 consultations (with nursing type 1 covering diabetes, child or maternal consultations, and type 2 covering consultations for vaccination and for other types of nursing treatments). Key differences between PHCCs and FHUs are summarized in Table 1. Each of these features was specifically taken into account when building the DES models.

**Table 1:** Key differences between PHCCs and FHUs programmed in the DES model

	PHCC	FHU
**Timetable**	Working days (8am - 8pm) Weekend (10am - 8pm)	Working days (8am - 8pm)

**Appointment scheduling**	Medical	Medical and nursing

**Medical consultations**	There are patients who are not registered with a GP	All patients are registered with a GP

**Emergency/acute consultations**	Specific timetable to attend these patients, and physicians exclusively allocated to the permanent health unit	Patients are seen by their own GP during FHU normal working hours

**Remuneration**	42 hours/week-based salary	35 hours/week-based salary plus variable incentives (B model)

### Case study and computational implementation

**Figure 2:**
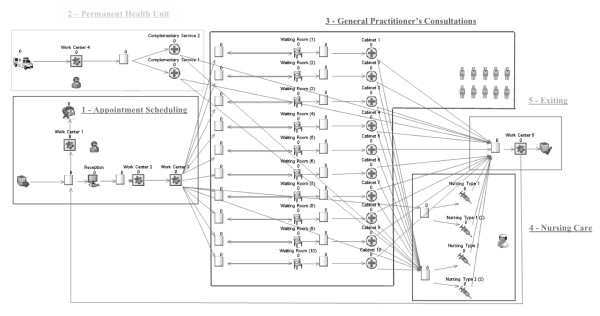
**Computational implementation for a single PHCC**.

The conceptual models for the PHCCs and FHUs were implemented using the Simul8 DES software [26]. The Simul8 software is a visual interactive modelling system for DES [23] that enables the creation of a computer model that replicates a process containing discrete entities and events occurring at discrete times. Figure 2 provides a screenshot of the Simul8 software showing, for a PHCC, patients' pathways, waiting rooms, doctors' cabinets, entry and exit points. The Simul8 software includes the Visual Logic programming language that was used to program the multiple features of PHCCs and FHUs. These features included units' opening times, doctors' working hours, and the routes to be followed by patients requiring different types of care.

The models were applied to a real case study including a convenience sample of nineteen primary health care units (thirteen PHCCs and six FHUs) from the Greater Lisbon area. This area comprises nine municipalities, and our work included units from three of these municipalities - Lisboa, Oeiras and Cascais. This is an urban and densely populated area, with a population growth rate above the national average [9], which places increased pressure on health care provision.

An individual DES model was implemented for each of the nineteen primary health care units. Public data from multiple sources was used to build the model for each FHU and PHCC, namely: (i) reports of the Portuguese Ministry of Health [7,17]; (ii) data from the Portuguese National Statistical Institute [27,28]; (iii) two reports commissioned by the Portuguese Health Economics Association [10,19], and (iv) specific information from local FHU reports [29-33]. In a few cases, where data was missing, data were obtained through direct contact with the units. In Tables 2 and 3 we show information on key parameters and probability distributions used in the models and on the performance indicators (PI) used to compare the FHUs with the PHCCs.

**Table 2:** List of key performance indicators used in the model

Indicator	Mainly informs on...
**Appointment scheduling**	

Number of days required to arrange a GP consultation	Accessibility

**Ambulatory consultations**	

Annual number of consultations per physician	Productivity (Efficiency)

Time spent in the waiting room (min)	Accessibility

**Emergency/acute consultations**	

Annual number of acute/emergency consultations per physician	Productivity (Efficiency)

Waiting time for an acute/emergency consultation (min)	Accessibility

**Nursing consultations**	

Annual number of nursing consultations per nurse	Productivity (Efficiency)

Time spent in the waiting room for diabetes, child or prenatal consultations (type 1) (min)	Accessibility

Time spent in the waiting room for vaccinations or other types of treatments (type 2) (min)	Accessibility

**Costs**	

Annual costs of diagnostic, medication and other treatments (€)	Costs (Efficiency)

Annual costs of professionals (€)	Costs (Efficiency)

Annual total costs (€)	Costs (Efficiency)

**Table 3:** List of the key parameters and probabilistic distributions used in the model

	Parameter	Value	Source of information
**Entry**	Inter-arrival time	Average distribution	Activity reports of each health care unit
		
	Type of consultation	Probability profile	
		
	Number of GPs	Predefined according to each unit	
	
	Days for the consultation	Predefined according to each unit	
		
	GP's schedule	Predefined according to each unit and respective GP	
		
**Internal flows**	Nurse's schedule	Predefined according to each unit and respective nurse	&
		
	Duration of adult consultations	Log normal distribution	data provided by health care units through their direct contact
		
	Duration of other consultations	Log normal distribution	
		
	Duration of nursing type 1 consultations	Average distribution	
		
	Duration of nursing type 2 consultations	Average distribution	

**Remuneration**	Cost per consultation with professionals	PHCC (€13.25) FHU (€16.32)	[10]
	
	Cost per consultation of diagnostic exams, drugs and other treatments	PHCC (€39.20) FHU (€29.20)	[10]

**Exit**	Patients' re-entry	Probability profile	Activity reports of each health care unit

The PIs described in Table 2 are process indicators related to the provision of primary care services that, using the taxonomy of Donabedian [34], capture two dimensions of quality in health care delivery: the ease with which persons can obtain care, i.e. accessibility; and the ability to lower the cost of care without diminishing attainable improvements in health, i.e. efficiency. Efficiency was further decomposed into productivity indicators - on the production per unit of input (for example, consultations per GP) -, and on the costs incurred to produce that amount (for example, total costs for delivering those consultations). One should note, however, that given the structure of the DES model, indicators of accessibility and productivity in Table 2 are related - for example, if each GP provides a higher level of consultations, not only his/her productivity increases, but also accessibility as measured by waiting times for consultations is expected to decrease.

The key parameters of the model (Table 3) were calibrated with real data from the year 2007 and included information regarding resources, production and operational costs for each unit. Given the variability and uncertainty associated with some parameters of the model, as shown in Table 3 several parameters are associated with a probability distribution. As a result, the outputs/PIs of the model are not point estimates but probability distributions. While some PIs produced by the DES model could be compared with real data, the model also generated new information for which real data was not available (for example, on waiting times). Because of difficulties in obtaining costs from FHUs, following the assumptions used by Gouveia et al. [10], we used cost data from the experimental units previously described, and that were in operation in 2005, to simulate FHU costs.

Each of the nineteen implemented models was independently run using a trial of five runs (i.e. using different sets of random numbers). As the number of runs directly affects the accuracy and the time required to run the model [35], a compromise was needed so as to run the models with good accuracy and within a reasonable amount of time, leading to the choice of five runs. Each trial of five runs for each primary care unit required, on average, two hours of processing, using an Intel^® ^CPU 1.60 GHz with 2.00 GB of RAM together with Simul8 13.0 and Microsoft Excel 2003 software. Each run included two time periods. First, a warm-up period was used so that demand, supply, queues and other parameters of the models could reach similar values to the real system. Following literature guidelines [35], a warm-up period of 52 weeks (one complete year) was found to be adequate. The remaining period was the results collection period that entailed a working year period of 50 weeks. Using the information from five runs, the results were returned in the form of 95% confidence intervals (CIs). According to Hauge and Paige [35], by ensuring that the 95% CIs returned by the models would enclose the real data relative to the year 2007, it was possible to confirm their validity and consequently use the models to simulate new cases (e.g. the conversion of a PHCC into a FHU). PIs presented in the form of CI should be interpreted as the result of variability and uncertainty in several inputs of the model, displayed in Table 3. The validation results for the total number of ambulatory consultations performed in a year are presented as an example in Table 4 (results per health care unit).

**Table 4:** Key data used for model validation (95% confidence intervals reported in brackets)

		Total number of medical consultations		
		
		Real	Model	Variation (%)
**Benfica**	PHCC Benfica	38464	[38356; 39579]	[-0.28; 2.90]
	
	PHCC Marchal Carmona	56609	[56948; 58011]	[0.6; 2.48]
	
	PHCC Carnide	26417	[25912; 26200]	[-1.91; -0.82]
	
	FHU Rodrigues Migueis	30065	[29676; 30238]	[-1.29; 0.58]

**Sete Rios**	PHCC Sete Rios	141671	[139235; 140607]	[-1.71; 0.75]
	
	FHU Tílias	26425	[24188; 25189]	[-8.47; -4.61]

**Carnaxide**	PHCC Linda-a-Velha	86720	[83899; 84913]	[-3.25; -2.08]
	
	PHCC Algés	54141	[53724; 54591]	[-1.27; 0.33]
	
	FHU Dafundo	33237	[33208; 33637]	[-0.08; 1.20]

**Oeiras**	PHCC Oeiras	83480	[78246; 79901]	[-6.27; -4.29]
	
	PHCC Paço de Arcos	70853	[67808; 69567]	[-4.30; -1.82]
	
	PHCC Barcarena	21783	[21696; 22109]	[-0.40; 1.47]
	
	FHU Delta	34042	[34135; 34574]	[0.27; 1.56]
	
	FHU São Julião	41186	[40538; 41164]	[-1.57; -0.05]

**Cascais**	PHCC Cascais	76881	[78160; 80415]	[1.66; 4.60]
	
	PHCC Estoril	61556	[61758; 63117]	[0.33; 2.54]
	
	PHCC Alvide	34636	[33785; 35599]	[-2.46; 2.78]
	
	PHCC Alcabideche	30631	[30883; 31094]	[0.81; 1.51]
	
	FHU Marginal	45198	[44115; 45088]	[-2.40; -0.24]

### Modelling the conversion of a PHCC into a FHU

Converting thirteen PHCCs into FHUs demanded for the outline of a set of assumptions regarding the legislation that defined FHUs features and the guidelines for FHUs creation (resources, composition, size, operating mode and financing rules) [36,37]. The following procedure was adopted: first, depending on its dimension, each PHCC was divided into smaller subunits with six to nine physicians, six to nine nurses and, on average, five administrative staff; each of these smaller subunits corresponded to an independent FHU. Second, in each of these subunits the permanent acute care unit was discontinued and GPs working in that unit were assigned exclusively for medical consultations in the FHU. In this way, as previously explained, acute cases started to be treated in the FHU regular working hours. Third, nursing care started to be scheduled, instead of patients showing up (randomly) for unscheduled nursing consultations. Fourth, remuneration of the health care professionals was changed from the salary without incentives to the model B remuneration system previously described. Finally, the demand from each PHCC was split between the corresponding FHUs, as a proportion of the GP resources allocated to each FHU.

## Results

Tables 5 and 6 present the PI values obtained for the PHCC and FHU organizational models, and changes in the PI values due to the conversion of the thirteen PHCCs into FHUs, respectively. Given the uncertainty and variability related with the estimation of several parameters of the DES model that lead to the use of the probability distributions described in Table 2 the PIs/outputs of the model (shown in Tables 5 and 6) are presented in the form of CIs.

**Table 5:** 95% confidence intervals for the PIs for the PHCCs and the FHUs, and percentage differences of averages

Indicator	PHCC	FHU	Difference between averages
**Appointment scheduling**			

Number of days required to schedule a medical consultation	[27; 39]	[14; 17]	**- 54%**

**Medical consultations**			

Annual number of consultations per GP	[3914; 4479]	[4197; 4672]	**+ 6%**

Time spent in the waiting room waiting for an ambulatory consultation (min)	[52; 81]	[27; 42]	**- 48%**

**Emergency/acute consultations**			

Annual number of emergency/acute consultations per physician	[488; 794]	[497; 802]	**+ 1%**

Time spent in the waiting room waiting for an acute/emergency consultation (min)	[6; 26]	[10; 15]	**- 21%**

**Nursing consultations**			

Annual number of nursing consultations per nurse	[2319; 2929]	[2145; 2856]	**- 4%**

Time spent in the waiting room waiting for a nursing type 1 consultation (min)	[5; 9]	[2; 5]	**- 50%**

Time spent in the waiting room waiting for a nursing type 2 consultation (min)	[7; 15]	[3; 10]	**- 42%**

**Table 6:** Results obtained after converting the PHCC into the FHU, for the whole sample of units (point estimates for costs and 95% confidence intervals for the remaining PIs, and percentage differences of averages)

Indicator	Before Conversion	After Conversion	Difference between averages
**Accessibility**			

Number of days required to schedule a medical consultation	[24; 34]	[14; 17]	**-45%**

Time spent in the waiting room waiting for a medical consultation (min.)	[48; 70]	[33; 43]	**-36%**

Time spent in the waiting room waiting for an acute/emergency consultation (min.)	[9; 21]	[12; 15]	**-12%**

Time spent in the waiting room waiting for a nursing type 1 consultation (min.)	[4; 8]	[3; 5]	**-38%**

Time spent in the waiting room waiting for a nursing type 2 consultation (min.)	[7; 13]	[5; 8]	**-38%**

**Productivity**			

Annual number of medical consultations per physician	[4037; 4463]	[4390; 4688]	**7%**

Annual number of nursing consultations per nurse	[2581; 2695]	[2589; 3169]	**9%**

Annual number of emergency/acute consultations per physician	[567; 799]	[616; 772]	**2%**

**Costs**			

Annual costs with professionals (1000000€) *(and with 10% increase in each type of cost, after the conversion, in italic)*	15 *(15)*	19 *(19)*	**25%** ***38%***

Annual costs with means of diagnostic, medication and other treatments per primary care unit (1000000€) *(and with 10% increase in each type of cost, after the conversion, in italic)*	41 *(41)*	35 *(34)*	**-16%** ***-7%***

Annual total costs (1000000€) *(and with 10% increase in each type of cost, after the conversion, in italic)*	57 *(57)*	54 *(53)*	**-5%** ***5%***

Results displayed in Table 5 show that the average number of days a patient had to wait for an appointment with a GP was 54% lower in the FHUs (33 vs. 15 days on average). The number of consultations by GP was 6% higher in FHUs, while a similar number of emergency/acute consultations were performed by GPs in each organizational model per year. Waiting times for emergency/acute consultations were on average shorter in FHUs, and the annual number of nursing consultations was slightly lower in FHUs. Finally, the average time spent in the waiting room for a nursing appointment was substantially lower in FHUs.

Changes in the PIs resulting from the conversion of the thirteen PHCCs into FHUs are shown in Table 6. We found a 45% reduction in the average number of days required to wait for a GP appointment (from an average of 29 to 16 days); a 36% reduction in the average time spent in the waiting room for medical consultations (from an average of 59 to 38 minutes); a 7% increase in the average number of medical consultations performed (from an average of 4250 to 4539 annual consultations per GP); a 9% increase in nursing appointments (from an average of 2638 to 2876 annual appointments per nurse); no significant changes in the average number of emergency/acute consultations; and a 25% increase in the costs of professionals, together with a 16% reduction in the costs of diagnostic exams, drugs and other procedures, leading to a 5% average reduction in total costs (from an average of €57 million to €54 million per year). When the costs of professional and the costs of diagnostic exams, drugs and other treatments per consultation are increased by 10% during the conversion from PHCCs into FHUs, total costs increase by 5%.

## Discussion

Limitations and assumptions used must be taken into account. All models are simplifications and are not able to represent the complexities of entire systems. The simulation models for PHCCs and FHUs do not inform about the impact of organizational reforms on several policy dimensions, such as implications on equity and responsiveness. Because of the complexity and time required to run each PHCC and FHU model, it was not possible to run all the PHCC and FHU models within an integrated and interacting multi-facility system, a feature strongly recommended for future models. Such a system would allow for modelling interactions between the activity of PHCCs and FHUs located in the same area - for instance, it could be tested the impact in the whole system of moving unregistered patients from a PHCC to GP lists from another FHU; and the impact of converting one PHCC into several FHUs when demand for the services provided in these units is jointly modelled. While this would be expected to change accessibility results, productivity results would be expected to maintain given the high use of available resources. Another limitation was the use of past data to simulate behaviour: some of the data from the FHUs used the first operational FHUs as a reference. However, these units were run by highly motivated GPs who embraced the FHU project and were not afraid of organizational changes. Our results might thus overestimate the gains because of these motivational effects, and the future conversion of PHCCs into FHUs may generate comparatively lower gains. Although motivational effects were clear during the implementation of the first FHUs, it can be argued that these effects will become less evident with time. In future studies self-selection issues in the creation of FHUs as time passes and more health care professionals become involved in FHUs should be evaluated. FHUs were assumed to have the same costs as the primary care units funded by the experimental system that operated in 2005 (following the assumptions used in [10]), and thus there might be an underestimation of costs for FHUs, in particular of costs with personnel. Sensitivity analysis shows that results on costs are very much dependent on the net effect of increased costs with professionals and decreased costs with diagnostic exams, drugs and other procedures, for which there is a margin for savings [6]. Finally, the study focused on urban PHCCs and FHUs. A similar study in rural areas could arguably produce different results because of different demographics, health care needs, level of health care resources [38], as well as because of lower geographic access to hospital services (it is hard to hypothesize how these aspects could possibly change our results).

Our results suggest that there are significant potential gains in converting PHCCs into FHUs in terms of accessibility and productivity. Regarding the scheduling of consultations, a 45% difference in the waiting time for a consultation in a FHU was found. The number of medical consultations performed by a GP per year was higher, on average, in FHUs, suggesting that, with the same resources, the FHU organizational model entails a higher productivity. This conversion might also result in a 7% increase in the number of medical consultations (from 4250 to 4539 annual consultations per GP), potentially contributing to an increase in the number of consultations delivered to registered patients and to a decrease in the number of patients not registered with a GP (for instance, through an increase in the number of registered patients per GP), which, according to the Institute of Informatics and Financial Management in Health [7], amounts to 16% of the population in the Greater Lisbon area. Potential gains to be achieved by converting PHCCs into FHUs are partly explained by: (i) contrarily to PHCCs, in FHUs almost all medical time is dedicated to clinical activity, as opposed to PHCCs, where six hours a week on average are allocated to administrative and/or management tasks, thus leading to less time for clinical work; (ii) in FHUs each GP sees mainly patients from his/her patient's list, and only for acute unscheduled consultations they see patients registered with other GPs from the same FHU; whereas GPs in PHCCs provide a much higher proportion of consultations to patients not belonging to their own list or for patients that are not registered with a GP; and (iii) the transfer of GP from acute care in PHCC to a normal working schedule in FHUs. However, one should note that these productivity gains may be attenuated in the future if differences in weekly working hours disappear, giving place to similar weekly schedule for every GP, regardless of the type of primary care organization. These results also may suggest that, despite well-known difficulties with group practice work in primary care [39], teamwork in FHUs contributes to higher productivity. These results are in line with previous studies which reported that there is scope for improving productivity and access of PHCCs [2].

No significant differences regarding emergency or acute cases were found between FHUs and PHCCs, meaning that the capacity for dealing with emergency situations was not compromised in an FHU-based model. Resources savings and improvements in accessibility can thus be expected in the FHU model regarding acute and emergency cases.

The results also show that the nursing appointments were 9% higher on average in a FHU. Setting up nursing appointments instead of the usual 'first comes first served' criterion seemed to allow nurses to carry out a higher number of consultations in the same time, thus contributing to gains in productivity.

Despite an increase in the number of medical consultations performed in FHUs, the results suggest that the time patients spend in the waiting room might, in fact, decrease. This may be explained by the use of empty slots between consultations, which, without loss in quality, allow emergency and acute situations to be dealt with, and also enable the system to accommodate variability in the duration of medical consultations. The results equally suggest that by having the nursing appointments previously set up, the waiting time of patients decreases, despite an increase in the number of appointments. It should be borne in mind that, despite gains in accessibility and productivity, adequacy and necessity of care were not addressed in this model.

The results on costs are in agreement with those previously described [10]. Although when converting PHCCs into FHUs there is an increase in the cost with professionals, spending on diagnostic exams, drugs and other procedures decreases, leading to a decrease in total costs. It is important to mention that the costs analysed in this work (salaries, diagnostic exams, drugs and other treatments) accounted, on average, for 90% of the total costs in these units [18,19] and, given limitations of the cost data in use, should be interpreted as rough approximations.

Gains from creating FHUs in Portugal are in line with the evaluation of primary care reforms in other countries. For example, in some provinces of Canada groups of physicians have been forming family health networks since 1998 [12], having contributed to improve accessibility of care to citizens [40]; successive reforms over the last 15 years in the English NHS that adopted new organizational forms (e.g. GP fundholding, Primary Care Trusts) and developed new pay-for-performance incentives, were shown to improve equity and quality of care [41].

## Conclusions

In summary, the FHU organization model has the potential to generate gains in productivity and accessibility in the Portuguese primary care sector, while not having a significant impact on costs. This study shows that DES models may be useful to model primary health care systems and for testing the impact of new reforms [42]. Although it is difficult to represent all the complexities of a primary care unit within a simulation model, appropriate simplifications may provide an answer to that problem. The right level of detail is critical in DES models [43] and a widely recognized guideline, that we followed, was to keep the model as simple as possible while capturing the necessary features of interest [22].

Future work might explore the application of the DES model on improved and more complete data (in particular on costs) and on PHCCs and FHUs located in other geographical areas with distinct features; running the models with new conditions (such as price changes); simulating the behaviour of several PHCCs and FHUs together in an integrated system, although this requires either simplifying the models or higher computational power; and analysing the implications on health outcomes.

## Abbreviations

CI: confidence interval; DES: discrete event simulation; ERR: experimental remuneration regimen; FHU: family health unit; GP: general practitioner; NHS: National Health Service; PHCC: primary health care centre; PI: performance indicator.

## Competing interests

ASF and MDO declare no conflicts of interest. ABS was a member of the Mission for Primary Health Care, the Ministry of Health task force for the launch and implementation of the primary health care reforms reported in this paper.

## Authors' contributions

ASF was responsible for the research process, data analysis and interpretation and for writing the paper. MDO and ABS were responsible for the conception and design of the study and helped in the data collection, analysis and interpretation, as well as in the writing of the paper. All authors read and approved the final manuscript.

## Pre-publication history

The pre-publication history for this paper can be accessed here:

http://www.biomedcentral.com/1472-6963/11/274/prepub
